# New Frontiers of microRNA in Heart Failure: From Clinical Risk to Therapeutic Applications

**DOI:** 10.3390/jcm14186361

**Published:** 2025-09-09

**Authors:** Isabella Fumarulo, Andrea De Prisco, Elia Nunzio Maria Salerno, Salvatore Emanuele Ravenna, Marcello Vaccarella, Barbara Garramone, Francesco Burzotta, Nadia Aspromonte

**Affiliations:** 1 Department of Cardiovascular Sciences, Fondazione Policlinico Universitario A. Gemelli IRCCS, 00168 Rome, Italy; isabella.fumarulo@guest.policlinicogemelli.it (I.F.); andrea.deprisco01@icatt.it (A.D.P.); elianunziomaria.salerno01@icatt.it (E.N.M.S.); marcello.vaccarella@policlinicogemelli.it (M.V.); barbara.garramone@policlinicogemelli.it (B.G.); francesco.burzotta@policlinicogemelli.it (F.B.); 2 Department of Cardiovascular Sciences, Catholic University of the Sacred Heart, 00168 Rome, Italy; 3 Department of Life Science, Health and Health Professions, Università degli Studi Link, 00165 Rome, Italy; emanueleravenna.md@gmail.com

**Keywords:** heart failure, microRNA, biomarkers, pathogenesis, diagnostics, therapeutic perspective

## Abstract

Heart failure (HF) is an increasingly prevalent disease with a major impact on morbidity and mortality worldwide. Continuous advancements in diagnostic and therapeutic strategies have significantly improved patient outcomes; however, precise biomarkers and novel therapeutic targets are still needed. In recent years, microRNAs (miRNAs) have emerged as promising biomarkers and potential therapeutic targets in HF. They consist of small, noncoding RNA molecules that regulate gene expression post-transcriptionally and are detectable in both tissues and blood, with disease-specific expression profiles that make them attractive candidates for non-invasive diagnosis, prognostic risk stratification, and even therapeutic interventions. In HF, miRNAs contribute to pathogenesis by modulating fibrosis, apoptosis, hypertrophy, and inflammation. The aim of this review is to analyze the role of circulating and tissue miRNAs in HF as biomarkers and as therapeutic targets. The future management of HF should include strategies to modulate miRNA expression in order to modify the disease trajectory and improve clinical outcomes.

## 1. Introduction

Heart failure (HF) represents the final pathway of several cardiovascular disorders, including ischemic heart disease, hypertension, valvular heart disease, and cardiomyopathies (hypertrophic cardiomyopathy (HCM), dilated cardiomyopathy (DCM), and restrictive cardiomyopathy (RCM)) [1,2], that ultimately lead to inadequate cardiac output for meeting the body’s oxygen needs or the elevated filling pressures needed to sustain it [3]. The result is a tough multisystemic syndrome, with symptoms and signs due to hypoperfusion and the congestion of several organs [4,5,6], and electrical issues due to cardiac conduction system dysfunction [7]. Despite advancements in medical and device therapies [8,9,10], HF progression is often unstoppable, reaching the end stages in a high percentage of patients [11], with a poor prognosis. Traditional biomarkers, such as B-type natriuretic peptide (BNP) and its N-terminal prohormone (NT-proBNP), are routinely used for diagnosis, prognosis, and treatment guidance [12]; however, they lack specificity. In this context, microRNAs (miRNA) have recently emerged as promising biomarkers and potential therapeutic targets in HF. MicroRNAs are endogenous, single-stranded, noncoding RNA molecules, long 22 nucleotides, which regulate gene expression post-transcriptionally by binding to complementary sequences on target messenger RNAs (mRNAs), leading to their degradation or translational repression [13]. In HF, miRNAs are involved in regulating cardiac remodeling, fibrosis, angiogenesis, apoptosis, and inflammation. Specific miRNAs have been identified in both myocardial tissue and circulating blood, correlating with HF severity, etiology, and prognosis. Notably, miRNAs are basically stable in biological fluids and are excellent for non-invasive testing. In addition to their utility in diagnosis, miRNA-targeted therapies, such as miRNA mimics or inhibitors (antagomiRs), are being developed and tested in preclinical models, showing an interesting potential to modify disease trajectories.

In the next paragraphs, we aim to provide a comprehensive overview of the current and emerging roles of miRNAs in HF, focusing on their diagnostic utility, prognostic relevance, and therapeutic perspectives.

## 2. Current Validated Biomarkers in Heart Failure

In standard clinical practice, biomarkers are employed by clinicians to support the diagnosis and prognostic assessment of heart failure.

Despite extensive research into various molecular biomarkers, natriuretic peptides continue to be the most commonly employed in the context of heart failure. They are, in fact, the sole biomarkers endorsed by the ESC in both the 2021 guidelines and the 2023 update [14].

Natriuretic peptides—atrial natriuretic peptide (ANP) and B-type natriuretic peptide (BNP)—along with their respective prohormones, mid-regional proANP (MR-proANP) and N-terminal proBNP (NT-proBNP), are released by cardiomyocytes in response to wall stress, originating predominantly from the atria for ANP and the ventricles for BNP [15,16]. As a result, their circulating levels directly reflect intracardiac pressure, the elevation of which is a key pathophysiological feature of heart failure.

The literature also describes additional cardiac biomarkers that, although less commonly used in clinical practice, reflect distinct pathophysiological mechanisms that complement those represented by natriuretic peptides (Table 1).

For instance, one of the most promising HF biomarkers is the sST-2, the soluble suppression of tumorigenesis-2. The ST-2 is a member of the family of IL-1 receptors and it is expressed by different organs like the eyes, lung, liver and heart [17]. sST-2 is a decoy receptor that antagonizes the anti-fibrotic and anti-remodeling effect of IL-33 binding the ST2 receptor [27]; its release from myocardiocytes depends on parietal sheer stress or inflammatory conditions [27]. As it is not heart-specific [18], it can only be used as a prognostic biomarker (PRIDE [28]).

One other promising biomarker is galectine-3. Galectins are a family of galactose-binding proteins involved in the regulation of proliferation and cellular differentiation [29].

Galectin-3, released by macrophages/fibroblasts during myocardial injury, reflects inflammatory–fibrotic remodeling and is associated with worse HF outcomes. Higher or rising levels (e.g., CORONA sub-analysis; ~17.8 ng/mL cut-points) predict rehospitalization/mortality; galectin-4 shows similar meta-analytic signals, supporting its use in multimarker risk stratification rather than in diagnosis [20,21,22,29,30].

Furthermore, high-sensitivity troponins were studied as alternative biomarkers to NT-proBNP in HF. Hs-cTns are historically known as myocardial damage biomarkers; their most common use is in myocardial infarction.

In HF conditions, the increase in intracameral pressure causes subendocardial chronic low-grade ischemia and myocardial apoptosis, which lead to the release of Hs-cTn that is not related to coronary artery disease [18,27].

Due to the established association between troponin release and myocardial injury, troponins have been evaluated as prognostic biomarkers in heart failure. Elevated circulating troponin levels have been consistently correlated with adverse clinical outcomes [19,28,29,31]. Nonetheless, the clinical application of troponins, sST2, and galectin-3 in heart failure management has not yet been standardized; as a result, natriuretic peptides remain the primary biomarkers routinely utilized in clinical practice.

## 3. The Mechanism of Action of microRNAs

MicroRNAs (miRNAs) are small endogenous noncoding RNAs, typically 19–25 nucleotides in length, that play a crucial role in regulating gene expression in the post-transcriptional phase [20]. Their discovery was revolutionary and changed our understanding of gene regulation, revealing that beyond simple transcriptional ON/OFF switches, there is a complex network of control [21].

**Figure 1:**
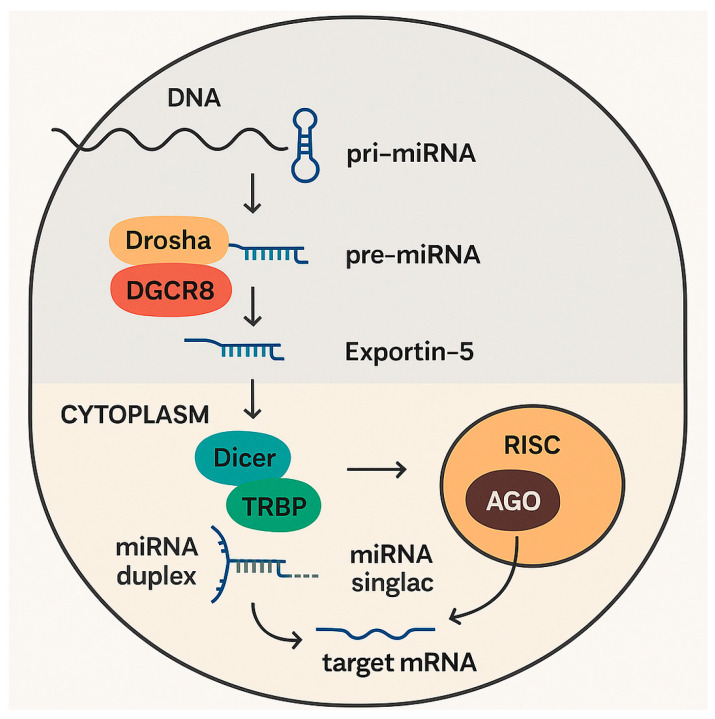
Production and mechanism of action of microRNAs.

Primary miRNAs (pri-miRNAs) are transcripted in the nucleus by RNA polymerase II or III; they are long, with hundreds to thousands of nucleotides, and present characteristic hairpin structures [22]. Subsequently, in a crucial and tightly regulated nuclear processing step, the RNase III enzyme Drosha (part of the microprocessor complex, along with its cofactor DGCR8 (DiGeorge syndrome critical region gene 8)) cleaves the pri-miRNA at the base of the hairpin, releasing a precursor miRNA (pre-miRNA) hairpin, which has about 70 nucleotides. The pre-miRNA is then exported from the nucleus to the cytoplasm by Exportin-5, a nuclear transport receptor, in a Ran-GTP-dependent process (Figure 1).

In the cytoplasm, the Dicer complex (another RNase III enzyme) cleaves the pre-miRNA hairpin, often in association with TRBP (TAR RNA-binding protein) and PACT (protein activator of interferon-induced protein kinase R). A short miRNA duplex is then generated, consisting of two imperfectly base-paired strands: the mature miRNA strand (guide strand), and the passenger strand (miRNA* or miRNA-star) [30].

After this process, one strand of the miRNA duplex, typically the less thermodynamically stable 5′ end, is preferentially loaded into the RNA-induced silencing complex (RISC). The RISC consists of a multi-protein complex with a core formed by an Argonaute (AGO) protein [32]. There are four AGO proteins in humans (AGO1, AGO2, AGO3, and AGO4), each with distinct roles and catalytic activities [23]. The selection of the guide strand and its loading into AGO is a critical phase. Then, the passenger strand is usually degraded, with infrequent exceptional cases, in which both strands can be functional.

Although, in most cases, the passenger strand is degraded, there are notable exceptions, in which both strands of the duplex are retained and are biologically active. These “dual-function” miRNAs can regulate distinct sets of target genes, thereby broadening the regulatory capacity of a single precursor. For example, both miR-126-3p and miR-126-5p exert functional effects in endothelial biology and angiogenesis, while miR-17-5p and miR-17-3p modulate different pathways involved in cardiac remodeling. The presence of two functional strands highlights an additional layer of complexity in post-transcriptional regulation, since dysregulation of either strand may contribute to the coordinated control of multiple HF-related signaling networks [24,25]

Once integrated into RISC, the mature miRNA searches for target mRNA molecules through sequence complementarity. The “seed” region of the miRNA, typically nucleotides 2–8 from the 5′ end, is the most important for target recognition based on base pairing. Perfect or near-perfect complementarity in this specific region allows for effective binding between mRNA and miRNA. However, additional base pairing in other different regions, particularly at the 3′ end of the miRNA, can contribute to the increased specificity and efficiency of binding [26].

The degree of complementarity between the miRNA and its target is pivotal in the subsequent steps. If the complementarity is perfect or near-perfect, the AGO protein (in humans, specifically AGO2 which possesses “slicer” activity) can cleave and degrade the target mRNA, leading to a rapid and irreversible silencing of gene expression. However, a perfect complementarity between miRNA and mRNA is more frequent in plants, while in animals, in most cases, the complementarity of miRNA–mRNA binding involves imperfect base pairing, particularly within the 3′ untranslated region (3′UTR) of the mRNA. In these cases, there is no direct mRNA cleavage. Instead, the primary mechanism of gene silencing becomes translational repression, which can be achieved by several underlying mechanisms [22].

RISC can interfere with the assembly of the translation initiation complex, for example, by blocking the binding of eukaryotic initiation factor 4E (eIF4E) to the mRNA cap, which can lead to the inhibition of translation initiation [32].

On the other side, miRNAs can induce ribosomes to disengage from the mRNA during elongation, causing premature termination of translation.

Furthermore, RISC can recruit enzymes, inducing deadenylation (the removal of the poly(A) tail) and decapping (the removal of the 5′ cap) of the mRNA, making it susceptible to exonucleolytic decay [32].

Finally, miRNA-targeted mRNAs can be sequestered into cytoplasmic processing bodies (P-bodies), which are sites where mRNAs are stored and undergo degradation, removing them from active translation.

In brief, miRNAs are small, endogenous, noncoding RNAs, essential in regulating gene expression in the post-transcriptional phases. Their mechanism of action is a complex multi-step process which starts with nuclear processing, continues with cytoplasmic maturation and RISC loading, and ultimately ends with targeting specific mRNAs, leading to their silencing through translational repression or degradation. Thus, miRNAs regulate gene expression in a multitude of biological processes, including disease pathogenesis and response to treatments, making them desirable as diagnostic biomarkers and therapeutic targets.

## 4. MicroRNAs as Emerging Biomarkers in HF: Diagnostic and Prognostic Utility

Although several biomarkers, such as natriuretic peptides, are used in the diagnostic process of HF, circulating miRNAs have been increasingly studied as new candidates for future diagnostic biomarkers in heart failure (Table 2). In fact, it is known that natriuretic peptides have high sensitivity for the diagnosis of HF; however, they show less specificity. Moreover, for differentiating heart failure with preserved ejection fraction (HFpEF) from HF with reduced EF (HFrEF), NT-pro BNP is less accurate. In this context, miRNAs can have an additive role in the diagnostic–therapeutic management of HF and its symptoms. With respect to breathlessness, a recent study showed how a specific miRNA, such as miR-423-5p, is differentially expressed between heart failure and patients with other causes of dyspnoea [33]. The study of the expression of miRNAs can be interesting in the analysis of patients with acute, rather than chronic, presentations. It seems that in patients with acute heart failure, there is a different expression of circulating miRNAs, including low levels of MiR-103, miR-142-3p, miR-30b, miR-342-3p, and high levels of miR-499 [34,35].

With respect to miRNa expression in different settings of HF, it is of interest that the miRNAs linked to several presentations, including increasingly worsening presentations, and worse and faster progressions, can be identifed. A recent meta-analysis tried to use these new RNA-based markers to stratify patients according to their risk. The analysis of more than five thousand patients showed a panel of four miRNAs (miR-27a-3p, miR-129-5p, miR 145-5p, miR-590-3p) that are predictable markers of all-cause death in patients with HFrEF. Moreover, miR-122-5p and miR-423-5p seem to be specifically linked with cardiovascular death in this group of patients. The study also revealed the role of miR-19a-3p as independent biomarkers in patients with HFpEF [38].

Several studies have provided details on validation cohorts and predictive performance. Ellis et al. analyzed a cohort of 225 patients with acute dyspnea, including >100 confirmed HF cases, and demonstrated that circulating miR-423-5p differentiated HF from non-cardiac causes with an area under the curve (AUC) of 0.91 and a median follow-up of 12 months [35]. Similarly, in their study, Zhang et al. enrolled nearly 200 patients with HFrEF and 100 controls, reporting after a median follow-up of 18 months that reduced circulating miR-19b levels predicted adverse outcomes, with a negative correlation to NT-proBNP [40]. A recent meta-analysis pooling >5000 patients with both HFrEF and HFpEF confirmed the independent prognostic value of miR-27a-3p, miR-129-5p, miR-145-5p, and miR-590-3p, with hazard ratios ranging from 1.5 to 2.0 for all-cause mortality, after a follow-up of up to 36 months [38]. We must note that most of the available evidence, while promising, is still based on modest sample sizes and relatively short-to-intermediate follow-ups, underscoring the need for larger, multicenter validation studies.

Other miRNAs, such as miR-122, have been implicated in the diagnosis of HFrEF. It is normally very abundant in the liver, where it regulates genes involved in cholesterol and fatty acid metabolism. It is known how this epigenetic marker is expressed in patients with HFrEF, reflecting liver damage due to chronic congestion. It seems that the upregulation of miRNA-122 can cause detrimental effects on the heart, with a noxious vicious cycle with liver damage. For this reason, this miRNA was found to be independently linked to all-cause mortality, even after adjustment for right ventricular dysfunction, cholinesterase, and liver injury [39].

Another recent study showed the role of another miRNA, miR-19-b, in patients with HFrEF. The study enrolled around 200 patients with HF and 100 controls; different comparisons of miR-19b were made in different groups. The study showed how patients with HFrEF had significantly lower miR19-b levels than the HFpEF and control groups. The decreased expression of this marker is associated with increased cardiac fibrosis, which is compulsorily linked to severe impairment of systolic ventricular function and to a worse prognosis. Moreover, the study remarked on the negative correlation between miR-19b and NT-proBNP (*p* < 0.001). However, miR-19b seems to not be useful in distinguishing healthy patients from those with HF. The association between miR-19b and NTproBNP is still more accurate for diferentiating between HFrEF and HFpEF patients [40].

More specifically, micro-RNAs can be very useful in the diagnostic evaluation of particular settings of patients with HF. For example, in patients with hypertrophic cardiomyopathy, a significant upregulation of miR29a was found. It appears that this specific biomarker can differentiate between hypertrophic obstructive cardiomyopathy, hypertrophic non-obstructive cardiomyopathy, senile amyloidosis, and aortic stenosis. Furthermore, this specific miRNA is positively related to the interventricular septum size and all remodeling processes [41].

With respect to cardiac amyloidosis, another study included patients with hereditary transthyretin mutation (ATTRm), patients with senile cardiac amyloidosis (ATTR wild-type), and patients with heart failure without amyloidosis. The study revealed significant high levels of miR-339-3p in ATTRwt compared to other cohorts [42]. Specifically, another biomarker, miR-150-5p, was found to be useful in differentiating ATTRm patients with typical symptoms, including neuropathy, from asymptomatic TTR carriers [43].

Another important study involved the prognosis and management of HF after myocardial infarction. In fact, several miRNAs are involved in impaired left ventricular contractility, remodeling, and the risk of death from heart failure. A recent work showed how cardiovascular death in patients with ischemic heart failure was associated with low levels of miR-126, while high levels of miR-508a-5p were notable in patients with non-ischemic heart failure [44].

Finally, the possibility of using miRNAs as a predictor for responses to therapeutic options is of interest. For patients with severe end-stage heart failure requiring left ventricular assist device (LVAD), a recent study showed that levels of circulating miR-1202 can predict responders from non-responder patients before LVAD implantation [45].

Similarly, in response to cardiac resynchronization therapy (CRT) a total of five miRNAs (miR-26b-5p, miR-145-5p, miR-92°-3p, miR-30e-5p and miR-29a-3p) were found to be higher in the livers of responding patients compared with those of non-responders [46].

Current ESC guidelines for the diagnosis and management of HF recommend the use of natriuretic peptides (BNP, NT-proBNP) as cornerstone biomarkers, with additional supporting roles for troponins, soluble ST2, and galectin-3 as prognostic tools [14]. While these molecules are well validated and integrated into routine clinical practice, they remain limited by suboptimal specificity, variability across patient populations, and poor discrimination between HF phenotypes (e.g., HFrEF vs. HFpEF). In contrast, circulating miRNAs have demonstrated disease-specific expression patterns, relative stability in biological fluids, and the potential to capture distinct pathophysiological processes such as fibrosis, hypertrophy, and inflammation. Notably, some miRNAs (e.g., miR-423-5p, miR-122, and miR-19b) have shown prognostic or phenotypic specificity beyond natriuretic peptides. However, translation into clinical practice requires large-scale validation, standardized assays, and the demonstration of added prognostic or therapeutic value when combined with established biomarkers. Thus, miRNAs should not be viewed as replacements but rather as complementary tools that may refine risk stratification and guide personalized therapy in future HF management frameworks.

## 5. Bench-to-Bedside Perspectives: Therapeutic Applications of microRNAs in HF

Some evidence has shown that more than half of RNA messengers are regulated by microRNAs, which play an important role in both physiological and pathological processes. MicroRNAs are known to be dysregulated in various cardiovascular conditions and could act as a potential biomarker for identifying these disorders. By regulating gene expression, they could also be involved in the maladaptive pathways of cardiomyocytes and represent a potential therapeutic target.

MicroRNA modulation could involve restoring the function of microRNAs, whose decrease is made detrimental by the administration of microRNA mimics. Conversely, microRNAs that are overexpressed could be inhibited using complementary RNA sequences called antagomirs.

Several microRNAs have been associated with heart failure in both animal models and clinical trials. Some of the therapies based on these data have been examined in humans; however, none have entered phase III trials (Table 3).

The overexpression of microRNA-132 (miRNA-132) is involved in cardiac remodeling, hypertrophy, and the progression of heart failure (HF). Inhibiting this pathway has been shown to significantly improve systolic and diastolic function in both ischemic and non-ischemic HF animal models [47,48]. Across preclinical studies, miR-132 emerges as a necessary and sufficient driver of pathological cardiomyocyte growth and adverse remodeling, making it a therapeutic target for heart failure [49]. In transgenic mice overexpressing the miR-212/132 cluster, weekly administration of antimiR-132 reversed hypertrophy, improved ejection fraction, reduced LV dilatation, and restored downstream targets such as FOXO3 and SERCA2a [50,51]. Mechanistically, antimiR-132 normalized action-potential duration, intracellular calcium handling, and sarcomere kinetics in isolated cardiomyocytes. In a large-animal post-MI model, two doses (day 3 intracoronary or intravenous, day 28 intravenous) at 1/5/10 mg·kg^−1^ improved EF (to ~44–45% with medium/high dosing), reduced LV end-systolic volume, interstitial fibrosis, and cardiomyocyte size, and lowered NT-proBNP versus placebo. Pharmacokinetic profiling showed comparable myocardial exposure after IV and IC delivery, an approximate cardiac tissue half-life of three weeks, and a strong inverse correlation between cardiac drug levels and functional miR-132 aligned with EF gains [50]. A chronic porcine post-MI HF model initiating therapy 1 month after infarction demonstrated that monthly IV CDR132L (5 mg·kg^−1^) for 3 or 5 months improved systolic function (+7–8% absolute vs baseline/placebo-corrected), attenuated LVESV progression, improved diastolic indices (dP/dt_min and EDPVR), reduced left-atrial volume, and lowered NT-proBNP, with sustained target engagement and no drug-related safety signals—thereby supporting a clinically practical monthly regimen [47]. CDR132L is a first-in-class, locked nucleic-acid antisense oligonucleotide that selectively inhibits cardiac microRNA-132-3p. Building on the aforementioned preclinical studies of miR-132 inhibition, the antisense oligonucleotide CDR132L has been investigated in phase 1 and 2 studies as a therapeutic candidate for heart failure. A study of 28 patients with stable heart failure confirmed that the administration of 10 mg/kg of CDR132L was safe and well tolerated in humans. In light of this evidence, the HF-REVERT trial was developed. This phase 2 trial aims to evaluate the efficacy and safety of CDR132L in patients with heart failure (HF) (FE < 45%) following an acute myocardial infarction (MI). The primary endpoint is the percentage change in left ventricular (LV) end-systolic volume index from baseline after six months, which is used as a marker of reverse remodeling. The main secondary endpoints are N-terminal pro-B-type natriuretic peptide (NT-proBNP) levels and quality of life. The study was completed in March 2025; results are yet to be published. Collectively, the findings may substantiate miR-132 inhibition as a means of preventing and potentially reversing adverse remodeling in HF; they will inform the planning of a follow-on outcomes trial testing CDR132L

Another trial (NCT04045405) will assess the safety, pharmacokinetics and pharmacodynamic parameters of CDR132L in patients with stable, ischemic heart failure (NYHA class I–III).

The ASTRAAS-HF (NCT04836182) is a phase 2 trial that will evaluate the effect an antisense inhibitor of angiotensinogen production (IONIS-AGT-LRX) on plasma angiotensinogen (AGT) concentration and N-terminal prohormone of B-type natriuretic peptide (NT-proBNP) levels in chronic heart failure participants with reduced ejection fraction (HFrEF, FE < 40%).

Other microRNAs, such as those related to fibrosis and hypertrophy (miT-133, miR-1 and miR-29) or angiogenesis (miRNA-92), have been analyzed in preclinical models and could play a role in heart failure [52]. MRG-110, an oligonucleotide that inhibits microRNA-92 (miRNA-92), was analyzed in a phase I trial. Modulating this angiogenesis-related pathway could play an important role in the development of ischemic heart failure [53].

MiR-200a was found to decrease significantly in ischemic heart tissue post MI. Recently [54], the restoration of its levels using agomiR-200a in a murine model of myocardial infarction was associated with an increase in the left ventricular ejection fraction of 68%, as well as a reduction in infarct size. The expression of miR-200a was inhibited due to H_2_O_2_ stress in MI cardiac tissues; however, the overexpressing of miR-200a could protect cells from death by regulating the Keap1/Nrf2 and β-catenin signal transduction pathways, as well as by reducing levels of IL-1β, IL-6 and TNFα.

microRNA-152 (miR-152) expression was upregulated in failing human hearts and in experimental animal models of heart failure (HF). Transgenic mice with cardiomyocyte-specific overexpression of miR-152 developed systolic dysfunction and dilated cardiomyopathy. Preclinical evidence indicated that inhibiting miR-152 preserved cardiac function in a pressure overload-induced HF model [55].

There are still challenges that limit the use of microRNAs in humans. One key challenge is the development of reliable and reproducible assays, as variability in sample processing and detection methods significantly impacts results across studies. In addition, a single microRNA (miRNA) can regulate many processes in different organs. On the other hand there is also redundancy in the microRNAs involved in a single biochemical and physiopathological pathway. Therefore, off-target effects and unpredictable biological consequences must be taken into account. Another hot topic concerns delivery. Although microRNAs are stable molecules, they must reach the correct tissue. In animal models, investigators often used local administration; however, systemic administration raises the question of how the drug will be delivered to the targeted tissue. Current delivery vehicles include liposomes, viral vectors (especially adeno-virus with cardiac tropism), nanoparticles and extracellular vesicles (EVs) [56]; however, these approaches are still associated with safety, immunogenicity, and scalability issues. Furthermore, cost-effectiveness and accessibility also need to be considered, particularly in resource-limited settings; it will be important to identify a specific subset of patients who can really benefit from these emerging therapeutic options. Finally, this is a very recent topic that is still evolving; rigorous multicenter validation trials will be necessary to meet the standards required for clinical approval.

## 6. Conclusions

Heart failure is a complex clinical syndrome burdened by high morbidity and mortality, requiring innovative approaches for diagnosis, prognostic assessment, and treatment. Natriuretic peptides still remain the cornerstone biomarkers in clinical practice; however, they lack specificity. Thus, novel molecular biomarkers have emerged. microRNAs appear to be promising biomarkers due to their key regulatory role in important pathophysiological mechanisms of heart failure, including fibrosis, hypertrophy, apoptosis, inflammation, and remodeling.

Circulating miRNAs show disease-specific expression patterns and are particularly stable in biological fluids; thus, they may be ideal candidates for non-invasive diagnosis and risk stratification. Specific miRNAs have been associated with different heart failure phenotypes (HFrEF vs. HFpEF), etiologies (ischemic vs. non-ischemic), disease stages (acute vs. chronic), and responses to therapies such as CRT and LVAD. Furthermore, several miRNAs seem to have prognostic value and may contribute to personalized treatment strategies.

The potential role of miRNA is not limited to diagnostics; it also involves the therapeutic field. In preclinical models, miRNA-based interventions using mimics or antagomiRs have demonstrated some efficacy. Clinical trials are ongoing; in a short time, we could witness a potential paradigm shift in heart failure management. However, significant issues remain, including targeted delivery, off-target effects, the redundancy of miRNA pathways, and cost-effectiveness. A major challenge in translating miRNA-based therapies into clinical practice is the development of safe and effective delivery systems. Current approaches include liposome-encapsulated oligonucleotides, viral vectors with cardiac tropism (e.g., adeno-associated viruses), nanoparticles, and extracellular vesicles; each offers distinct advantages in terms of stability, tissue targeting, and immunogenicity. Optimizing these carriers will be essential to achieve organ-specific delivery while minimizing off-target effects. Equally important is patient stratification: not all patients with HF may benefit from miRNA-targeted interventions in the same way. Future research should focus on identifying patient subgroups with the specific genetic backgrounds or circulating miRNA profiles that are most likely to respond. A precision medicine approach is needed, integrating conventional risk models with molecular analysis, with the objective of maximizing therapeutic benefit, reducing costs, and limiting unnecessary exposure to novel therapies.

In conclusion, miRNAs represent a promising frontier in heart failure, with potential clinical applications both in diagnostics and therapeutics. Future research should focus on validating miRNA panels in larger cohorts, optimizing delivery systems, and integrating these tools into precision medicine frameworks to improve outcomes in heart failure patients.

## Figures and Tables

**Table 1:** Biomarkers in HF.

Biomarker	Family	Source	Stimulus/Pathophysiology	Clinical Role	Representative Cut-Off	Key References
Atrial natriuretic peptide (ANP)	Natriuretic peptide	Atrial cardiomyocytes	Wall stress → release; reflects elevated intracardiac (atrial) pressure	Diagnostic and prognostic	Not standardized in ESC HF guidance	[14]
Mid-regional proANP (MR-proANP)	Natriuretic peptide (prohormone)	Atrial cardiomyocytes	Wall stress; stable surrogate of ANP release	Diagnostic and prognostic	Chronic > 40; Acute > 120	[14]
B-type natriuretic peptide (BNP)	Natriuretic peptide	Ventricular cardiomyocytes	Wall stress → release; reflects elevated intracardiac (ventricular) pressure	Diagnostic and prognostic	Acute HF > 100 pg/mL; Chronic HF > 35 pg/mL (ESC 2021 [14])	[15]
N-terminal proBNP (NT-proBNP)	Natriuretic peptide (prohormone)	Ventricular cardiomyocytes	Wall stress; stable surrogate of BNP release	Diagnostic and prognostic	Acute HF > 300 pg/mL; age-specific chronic HF cut-offs (ESC 2021 [14])	[15]
Soluble ST2 (sST2)	IL-1 receptor family (decoy receptor)	Cardiomyocytes, lung, liver, others	Shear stress and inflammation → release; antagonizes IL-33/ST2 protective pathway, promotes fibrosis	Prognostic (not diagnostic)	>20 ng/mL (PRIDE trial)	[17,18,19]
Galectin-3	Galactose-binding lectin	Activated macrophages and damaged cardiomyocytes	Inflammation-induced expression; drives fibrosis and ventricular remodeling	Prognostic (rehospitalization, mortality)	≥17.8 ng/dL (CORONA sub-analysis)	[20,21,22]
High-sensitivity cardiac troponins (hs-cTnI/T)	Cardiac structural protein	Cardiomyocyte cytosol and sarcomere	Myocyte injury/apoptosis due to chronic subendocardial ischemia and pressure overload (non-ACS)	Prognostic (severity and outcomes)	Results expressed in ng/L; prognostic thresholds vary by assay	[23,24,25,26]

**Table 2:** MicroRNAs as potential HF biomarkers.

miRNA	Diagnosis/HF Subtype	Regulation	Authors	Sample Size	Follow-Up	Predictive Value
miR-103	Acute HF	Decreased	Ellis et al. [35]	n = 225 (HF ~100 cases)	12 months	AUC ~0.85 (diagnosis)
miR-142-3p	Acute HF	Decreased	Ellis et al. [35]	n = 225	12 months	Prognostic, associated with mortality
miR-30b	Acute HF	Decreased	Ellis et al. [35]	n = 225	12 months	Prognostic, associated with readmission
miR-342-3p	Acute HF	Decreased	Ellis et al. [35]	n = 225	12 months	Prognostic accuracy for outcomes
miR-499	Acute HF	Increased	Corsten et al. [34]	n = 102 (AMI + HF)	30 days	Early diagnostic marker for cardiomyocyte injury
miR-423-5p	Acute HF	Increased	Tijsen et al. [33]	n = 225	12 months	AUC 0.91 for HF diagnosis
miR-190a	Chronic HF	Decreased	Wong et al. [36]	n = 156 HF, 80 controls	24 months	Correlated with LV remodeling
miR-210	Chronic HF	Increased	Feng et al. [37]	n = 120 HF, 60 controls	24 months	Linked with hypoxia, worse prognosis
miR-145-5p	HFrEF	Increased	Parvan et al. [38]	n = 5000+ pooled	Up to 36 months	HR 1.5–2.0 for mortality
miR-590-3p	HFrEF	Increased	Parvan et al. [38]	n = 5000+ pooled	Up to 36 months	HR ~1.7 for mortality
miR-129-5p	HFrEF	Increased	Parvan et al. [38]	n = 5000+ pooled	Up to 36 months	Prognostic value validated
miR-27a-3p	HFrEF	Increased	Parvan et al. [38]	n = 5000+ pooled	Up to 36 months	Prognostic value validated
miR-19a-3p	HFpEF	Increased	Parvan et al. [38]	n = 5000+ pooled	Up to 36 months	Independent biomarker in HFpEF
miR-122	HFrEF + congestion	Increased	Bonaventura et al. [39]	n = 200	12 months	Associated with hepatic congestion, adverse outcomes
miR-19b	HFrEF, prognosis	Decreased	Zhang et al. [40]	n = 200 HF, 100 controls	18 months	Correlated with NT-proBNP, adverse events
miR-29a	HCM	Decreased	Derda et al. [41]	n = 100 HCM	24 months	Linked with fibrosis markers
miR-339-3p	ATTRwt	Increased	Derda et al. [42]	n = 50 ATTRwt	12 months	Diagnostic marker
miR-150-5p	ATTRm	Increased	Vita et al. [43]	n = 40 ATTRm	12 months	Diagnostic marker
miR-126	Ischemic HF	Decreased	Qiang et al. [44]	n = 120 ICM patients	12 months	Correlated with endothelial dysfunction
miR-508a-5p	Non-ischemic HF	Increased	Qiang et al. [44]	n = 120 NICM patients	12 months	Correlated with adverse remodeling

HF = heart failure, HFrEF = heart failure with reduced ejection fraction, HFpEF = heart failure with preserved ejection fraction, HCM = hypertrofic cardiomiopathy, ATTRwt = wild-type transthyretine amilodosis, ATTRm = hereditary transthyretine amyloidosis.

**Table 3:** Clinical trials about potential microRNAs target therapies in HF. ↓: decrease; AGT: Angiotensinogen; AntimiR: anti-microRNA; MI: myocardial in; LVEF: left ventricle ejection fraction; farction; NTproBNP: N-terminal pro-B-type natriuretic peptide.

Trial	Phase	Target MiRNA	Delivery Method	Population	Outcome
HF-REVERT (CDR132L)	Phase II	miR-132	AntimiR (oligonucleotide)	Post-MI HF patients (LVEF < 45%)	Improved LV remodeling (↓LVESVi), ↓NT-proBNP, QoL benefit
NCT04045405	Phase I	miR-132	AntimiR (CDR132L)	Stable ischemic HF	Safe, well tolerated
ASTRAAS-HF	Phase II	miR-related AGT pathway	Antisense inhibitor (IONIS-AGT-LRX)	Chronic HFrEF (LVEF < 40%)	↓Plasma AGT, ↓NT-proBNP (ongoing)
MRG-110	Phase I	miR-92a (angiogenesis)	AntimiR	Ischemic HF	Target engagement, safety

## Data Availability

There are no new data associated with this article.
